# Late presentation of HIV positive adults and its predictors to HIV/AIDS care in Ethiopia: a systematic review and meta-analysis

**DOI:** 10.1186/s12879-019-4156-3

**Published:** 2019-06-17

**Authors:** Getaneh Mulualem Belay, Aklilu Endalamaw, Amare Demsie Ayele

**Affiliations:** Department of Pediatrics and Child Health Nursing, School of Nursing, College of Medicine and Health Sciences, University of Gondar, P.O. BOX: 196 Gondar, Ethiopia

**Keywords:** Ethiopia, HIV/AIDS care, Late presentation, Meta-analysis

## Abstract

**Introduction:**

Late presentation to HIV/AIDS care which is attended by problems like, poor treatment outcomes, early development of opportunistic infections, increased healthcare costs, and mortality is a major problem in Ethiopia. Although evidences are available on the prevalence and associated factors of late presentation to HIV/AIDS care, discrepancies among findings are appreciated. Thus, the country has faced difficulties of having a single estimated data.

**Objective:**

This study aimed to estimate the pooled prevalence of late presentation of HIV positive adults to HIV/AIDS care and its predictors in Ethiopia.

**Method:**

We searched all available articles through Google Scholar, PubMed, Web of Sciences, and EMBASE databases. Additionally, we accessed articles from the Ethiopian institutional online research repositories and reference lists of included studies. We included cohort, case- control, and cross-sectional studies in our review. Besides, we utilized the weighted inverse variance random-effects model. The total percentage of variation among studies due to heterogeneity was determined by *I*^*2*^ statistic. Searching was limited to studies conducted in Ethiopia and published in the English language. Publication bias was checked by Egger’s regression test.

**Results:**

A total of 8 studies with 7, 568 participants were included. The pooled prevalence of late presentation to HIV/AIDS care was 52.89% (95%CI: 35.37, 70.40). The odds of late presentation to HIV/AIDS care of frequent alcohol users [3.67(95% CI = 1.52–5.83)], high fear of stigma [3.90 (95% CI = 1.51–6.28)], chronic illness [3.34(95% CI = 1.52–5.16)], and the presence of symptoms at the time of HIV diagnosis [3.06 (95% CL = 1.18–4.94)] were higher compared to participants who did not experience the preceding.

**Conclusion:**

The prevalence of late presentation of HIV positive adults to HIV/AIDS care was high in Ethiopia. Frequent alcohol use, high fear of stigma, chronic illness, and the presence of symptoms at the time of HIV diagnosis were associated with high odds of late presentation to HIV/AIDS care.

**Trial registration:**

Registered in PROSPERO databases with the registration number of CRD42018081840.

**Electronic supplementary material:**

The online version of this article (10.1186/s12879-019-4156-3) contains supplementary material, which is available to authorized users.

## Introduction

Late presentations to Human Immunodeficiency Virus/Acquired Immunodeficiency Syndrome (HIV/AIDS) care is defined as persons presenting for care with a CD4 cell count below 350 cells/μl or presenting with an AIDS-defining event, regardless of the CD4 cell count [1].

HIV is a global pandemic health problem which affects all segments of the population. In 2017, around 36.9 million people were living with HIV in the world of which 21.7 million people accessed antiretroviral therapy [2]. The proportion of adults living with HIV and accessed ART (antiretroviral therapy) in the Middle East and North Africa was 29% [2].

Despite the accessibility of ART, different studies conducted in developed [3–6] and developing [7, 8] countries revealed that late presentation to HIV/AIDS care was a major problem in different countries. It was reported that in Sub-saharan Africa, over one-third of the HIV infected individuals presented to HIV/AIDS care late [9].

Accordingly, adults who presented lately to HIV/AIDS care encountered many problems, like poor treatment outcomes, increased mortality [3], high healthcare costs [10], and development of opportunistic infections [3]. Even though different strategies, like frequent change of ART treatment guidelines, extensive education about the management of HIV/AIDS, free testing, and treatment were delivered, late presentation is still a problem in Ethiopia.

Many efforts were made to determine the prevalence and associated factors of late presentation of HIV positive adults to HIV/AIDS care in Ethiopia. However, discrepancies among studies have made the acquisition of a single representative data difficult in Ethiopia. Therefore, this systematic review and meta-analysis aimed to estimate the pooled prevalence of late presentation of HIV positive adults to HIV/AIDS care and its predictors.

## Method

### Protocol registration

The protocol of this systematic review and meta-analysis has been registered in the International Prospective Register of Systematic Reviews (PROSPERO) with a registration number of CRD42018081840.

### Reporting

The Preferred Reporting Items for Systematic reviews and meta-analysis (PRISMA) guideline was used to report the results of this systematic review and meta-analysis [11] (Additional file 1).

### Databases and searching strategies

Google Scholar, PubMed, Web of Sciences, and EMBASE were used for searching all available articles. Additionally, we searched articles using the reference lists of included studies. We also accessed the Ethiopian institutional online research repositories using the following searching terms: “late presentation”, “delayed presentation”, “advanced stage presentation”, “late-stage presentation”, “Human Immune Deficiency Virus care”, “Human Immune Deficiency Virus/Acquired Immune Deficiency Syndrome care”, “HIV care”, “HIV/AIDS care”, “associated factors”, “predictors”, “determinants”, “risk factors”, “HIV positive individuals”, “HIV”, “adults”, and “Ethiopia”. The searching string was developed using “OR” and/or “AND” Boolean operators. Search details for PubMed databases were illustrated (Additional file 2). We conducted the search until 24 July, 2018.

### Inclusion and exclusion criteria

Articles included met the following criteria: [1] observational studies, including cohort, case-control and cross-sectional, [2] published and unpublished studies at any time, [3] being conducted in Ethiopia in the English language, and [4] studies that reported prevalence/or predictors of late presentation to HIV/AIDS care. However, conference papers, editorials, trials, reviews, program evaluations, and qualitative studies were excluded.

### Outcome measurement

Out of the included studies, three [12–14] were defined late presentation as people living with HIV/AIDS and had CD4 count 350cells/mm3 or World Health Organization (WHO) clinical stage III or IV during their first clinical visit for HIV care, four [15–18] were defined late presentation as people with WHO stage 3 or 4 irrespective of CD4 lymphocyte count or a CD4 lymphocyte count of less than 200/μl irrespective of clinical staging at the first visit for HIV/AIDS care, and one [19] was defined late presentation as HIV/AIDS patients registered with CD4 cell counts of < 100/ml.

In this study, chronic illness was defined as disease that needs three or more months of treatment with specified lists of diseases [20].

### Study selection and quality assessment

Primarily, all retrieved studies were imported to Endnote version 7 citation manager. Consequently, duplicated studies were carefully removed from Endnote. Then, two independent authors screened and assessed the titles and abstracts and reviewed the full texts. Any disagreement was solved through discussion and communication with the primary authors of the studies. After the full-text review, two investigators assessed the quality of the studies using the Joanna Brigg’s Institute (JBI) quality appraisal criteria adapted for cohort and case-control studies independently [11].

We used the following items to critically appraise studies. For cohort studies, we employed, [1] similarity of groups, [2] similarity of exposure measurement, [3] validity and reliability of measurement, [4] identification of confounders, [5] strategies to deal with confounders, [6] appropriateness of groups/participants at the start of the study, [7] validity and reliability of outcomes measured, [8] sufficiency of follow up time, [9] completeness of follow-up or descriptions of reasons for loss to follow-up, [10] strategies to address incomplete follow-ups, and [11] appropriateness of statistical analysis. For case-control studies we utilized, [1] comparable groups, [2] appropriateness of cases and controls, [3] criteria to identify cases and controls, [4] standard measurement of exposure, [5] similarity in measurement of exposure for cases and controls, [6] handling of confounders, [7] strategies to handle confounders, [8] standard assessment of outcome, [9] appropriateness of duration for exposure, and [10] appropriateness of statistical analysis. Studies were considered as low risk whenever fitted to 50% and/or above quality assessment checklist criteria were included in this systematic review and meta-analysis.

### Data extraction

Two independent authors extracted the data. Any disagreements between the authors were solved by discussion and consensus. Consequently, the first author of the study, year of publication, study area, design, population, sample size, prevalence of late presentation to HIV/AIDS care and/or identified associated factors were extracted. For associated variables reported in the primary studies, the AOR was extracted because of its importance for having adjusted and/or controlled possible confounders.

### Data analysis

To estimate the pooled prevalence of late presentation to HIV/AIDS care and pooled AOR of identified variables, we used the weighted inverse variance random-effects model. We assessed the percentages of total variations across studies using I^2^ statistics. The values of I^2^, 25, 50, and 75% represented low, moderate, and high heterogeneity, respectively. Publication bias across studies was checked using Egger’s regression test. A Stata version 11 (Stata Corp, college station, TX, USA) statistical software was used for all statistical analysis.

## Results

### Searching results

**Fig. 1:**
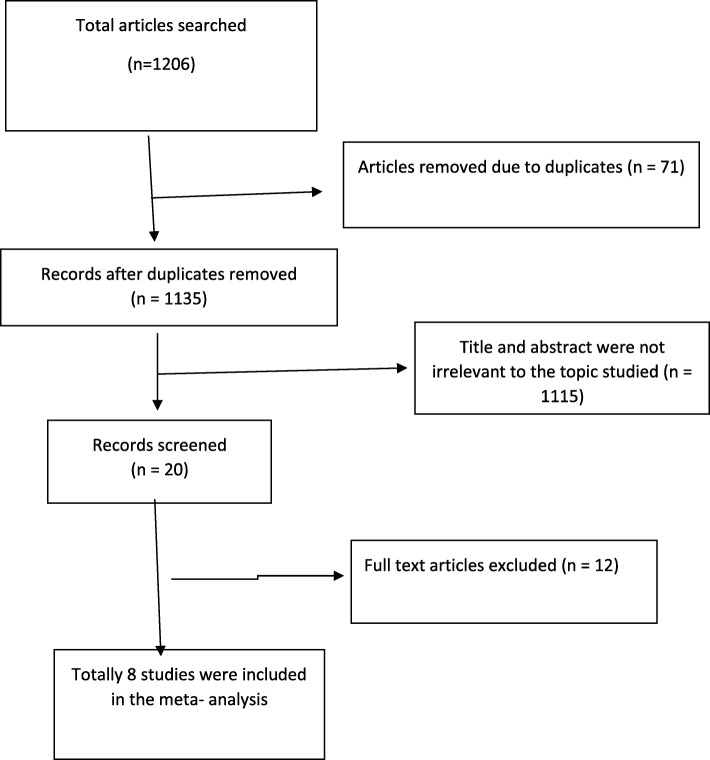
A PRISMA flow diagram of articles screening and process of selection

On the whole, 1206 citations were searched using different electronic databases of which 1133 articles were found from PubMed, 60 from Google scholar, 5 from EMBASE, 2 from the web of science, 5 from reference lists of included studies, and 1 from the Ethiopian research repositories. At the beginning, 71 articles were removed due to duplicates and 1115 due to irrelevant titles and abstracts. After a full-text review of 20 articles, 7 were removed due to study areas and 5 due to study designs. Finally, 8 articles were included in this systematic review and meta-analysis (Fig. 1).

### Characteristics of included studies

A total of eight studies [12–19] with 7568 participants were included in this study. Out of these studies, four [12, 15, 16, 19] were conducted through the case-control study design and the other four [13, 14, 17, 18] were retrospective cohort. Regarding study areas, three studies [15, 17, 18] were conducted in Oromia, two [13, 14] in Southern Nations, Nationalities, and Peoples’ Region (SNNPR), one [16] in Amhara, one [12] in Tigray, and one [19] in Harari. Detailed characteristics of included studies were described in Table 1.

**Table 1:** General characteristics of the included studies

Author	Region	Study design	Study population	Sample size	P (%)	Quality	Quality assessment result (%)
Gesesew H et al./2018 [17]	Oromo	Retrospective cohort	Adults	4900	67.7	low risk	72.73%
Abdu z etal/2014 [14]	SNNPRS	Retrospective cohort	Adults	714	34.4	low risk	63.6%
Gebru T etal/2018 [13]	SNNPRS	Retrospective cohort	Adults	320	50.5	low risk	54.6%
Gesesew/2016 [18]	Oromo	Retrospect cohort	Adults	289	59.9	low risk	63.6%
Abaynew y etal/ 2011 [16]	Amhara	case-control	Adults	320	not applicable	low risk	70%
Gesesew HA etal/2013 [15]	Oromo	case-control	Adults	309	not applicable	low risk	60%
Gelaw YA, etal/2015 [12]	Tigray	case-control	Adults	442	not applicable	low risk	70%
Asrat A/2010 [19]	Hareri	case-control	Adults	274	not applicable	low risk	60%

### Quality of included studies

Out of 8 studies, four [13, 14, 17, 18] were assessed using the JBI checklist for cohort studies and four [12, 15, 16, 19] were assessed using the JBI checklist for case-control studies. None of the studies were excluded after quality assessment.

### Meta- analysis

Publication bias was not observed among the included studies according to an Egger’s regression test assessment (*p*-value = 0.367).

### Qualitative description

Out of 8 studies, four were considered to determine the pooled prevalence of late presentation to HIV/AIDS care. However, all included studies were considered to determine the predictors of late presentation.

### Prevalence of late presentation to HIV/AIDS care

**Fig. 2:**
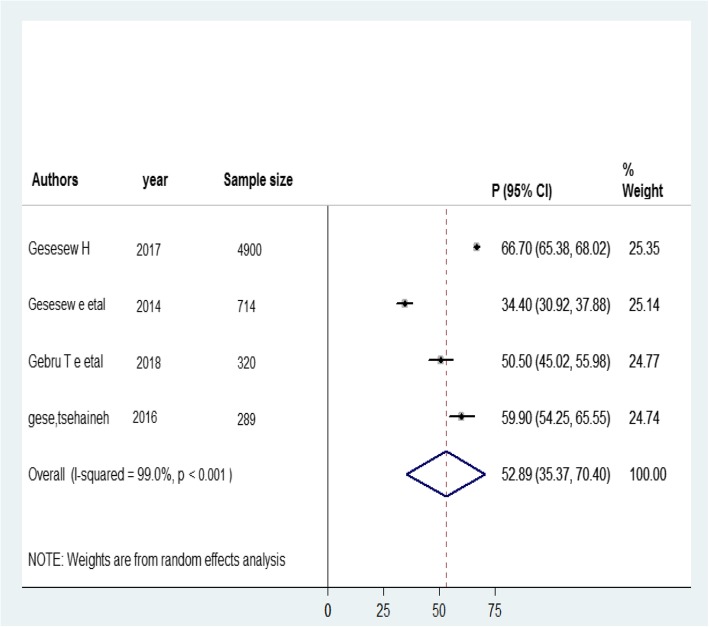
Forest plot of the prevalence of late presentation for HIV/AIDS care with 95% Cl. The midpoint and length of each segment indicated the prevalence and 95% confidence interval. The diamond shape revealed the pooled prevalence

The prevalence of late presentation to HIV/AIDS care ranged from 34.4% in SNNPR [14] to 67.7% in Oromia [17]. To estimate the pooled prevalence, four studies [13, 14, 17, 18] were used. Consequently, the pooled prevalence of late presentation to HIV/AIDS care in Ethiopia was 52.89% (95% CI: 35.37, 70.40; I^2^ = 99%; *P*-value < 0.001) (Fig. 2).

### Predictors of late presentation to HIV/AIDS care

In this systematic review and meta-analysis, predictors were categorized into three thematic areas like, socio-demographic, clinical and treatment-related predictors.

### Socio-demographic related factors

**Fig. 3:**
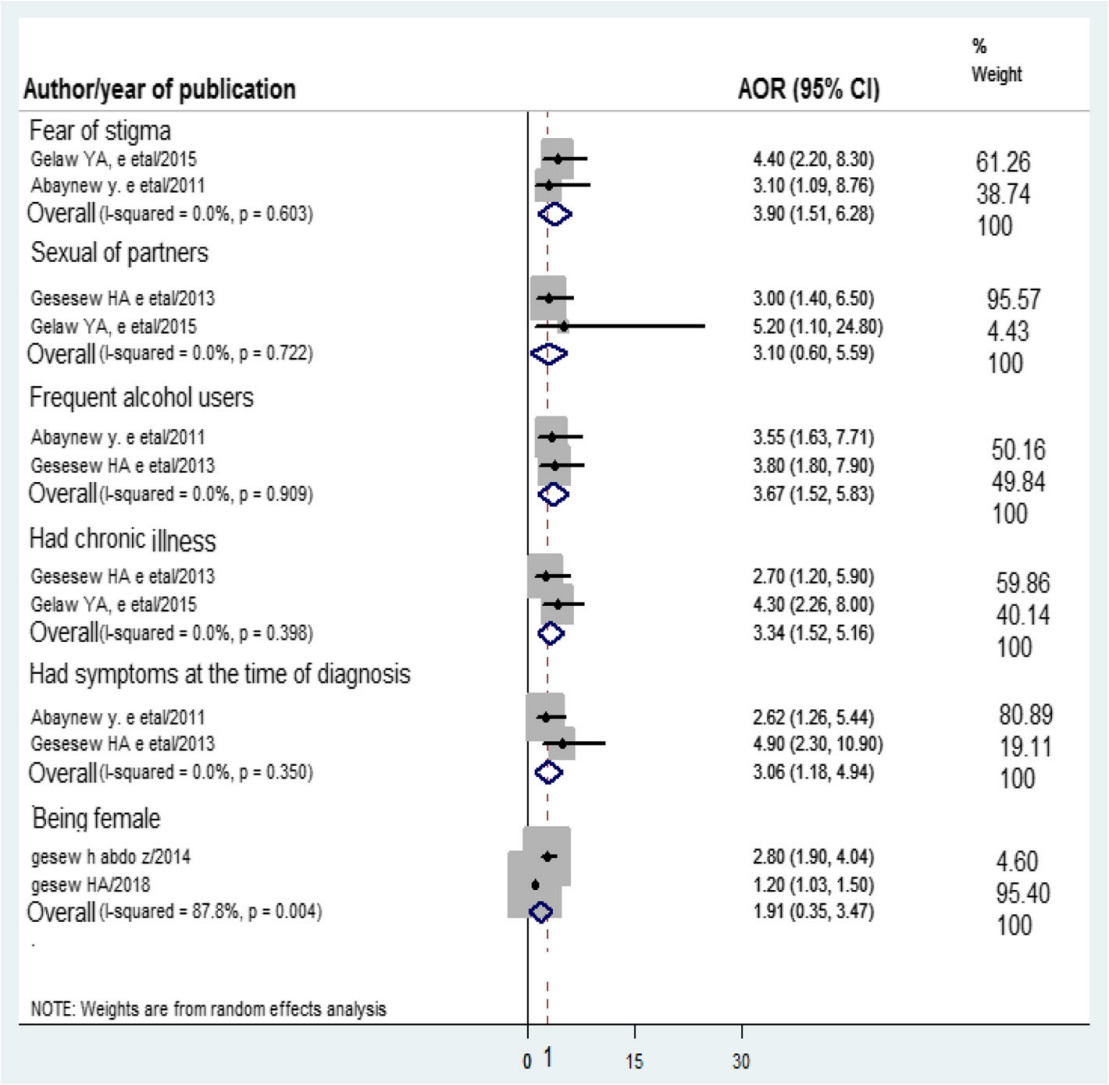
Forest plot of AOR of predictors of late presentation for HIV/AIDS care. The midpoint and the length of each segment indicated AOR and 95% CI respectively. The diamond shape showed the pooled AOR

Two studies [14, 17] reported that female sex was significantly associated to late presentation to HIV/AIDS care. On the contrary, one study reported that male respondents were 7.19 times more likely to present late to HIV/AIDS care than females 7.19 (95% CI: 1.279–8.447) [19]. However, in this systematic review and meta-analysis, the pooled AOR of late presentation was not significantly associated the female sex (Fig. 3).

Two studies [15, 16] showed that frequent alcohol use was a factor associated late presentation to HIV/AIDS care. The pooled AOR of late presentation to HIV/AIDS care among frequent alcohol users versus no alcohol users was 3.67(95% CI: 1.52, 5.83) (Fig. 3). Additionally, two studies [12, 15] reported that two or more sexual partners were associated with late presentation to HIV/AIDS care. However, the pooled result revealed that it is not significantly associated with the outcome variable (Fig. 3).

### Clinical-related factors

Two studies [12, 16] reported that there was an association between the fear of stigma and late presentation to HIV/AIDS care. The pooled AOR of late presentation to HIV/AIDS care among adults with high fear of stigma was 3.90 (95% CI: 1.51, 6.28) compared to adults with low fear (Fig. 3).

Two studies [15, 16] noted that the presence of symptoms at the time of HIV diagnosis was significantly associated with late presentation to HIV/AIDS care. The pooled AOR of late presentation to HIV/AIDS care among those who had symptoms at the time of HIV diagnosis versus those who had no symptoms at the time of HIV diagnosis was 3.06 (95% CI:1.18,4.94) (Fig. 3).

### Treatment-related factors

Two studies [12, 15] observed that HIV positive individuals who had chronic illness were significantly associated with late presentation to HIV/AIDS care. The pooled AOR of late presentation to HIV/AIDS care among those with chronic illness versus those who had no chronic illness was 3.34(95% CI: 1.52, 5.16) (Fig. 3).

## Discussion

In this work, the pooled prevalence of late presentation to HIV/AIDS care in Ethiopia was 52.89% (95% CI: 35.37, 70.40). High fear of stigma, frequent alcohol use, symptoms at the time of HIV diagnosis, and the presence of chronic illnesses were significantly associated with the problem.

This result is in line with those of studies conducted in Canada (46%) [4], Brazil (69.8%) [21], France (47.7%) [22], and Central Haiti (65%) [23] but lower than those of studies conducted in Cameron (89.7%) [24], South Africa (79%) [25], Benin (84.4%) [26], Asia (72%) [27],and Georgia (71.1%) [28]. This might be due to the availability of nation-wide health extension programs that help to create awareness about early diagnosis, enrolment, and treatment through community conversations, scaling up benchmark activities, and regular home visits.

On the other hand, the finding of this study was higher than that of a study conducted in West Africa (23%) [29]. The possible explanation for the variation might be the huge number of rural population with low education which leads to resistance to new health information.

Regarding predictors, fear of stigma was significantly associated with late presentation to HIV/AIDS care. The odds of HIV positive adults with high fear of stigma were nearly four times more likely to present late to HIV/AIDS care compared to their counterparts. This finding was in line with that of a systematic review and meta-analysis done in low and middle-income countries [30] and Zimbabwe [31]. This could be due to the fact that AIDS stigma affects social interaction and preventive behaviors, like healthcare seeking and HIV testing. Moreover, stigma results in social isolation that prevents people from getting HIV/AIDS related information.

This study revealed that late presentation to HIV/AIDS care among frequent alcohol user adults were nearly 4 times more likely compared to non-frequent alcohol users. This finding is supported by studies conducted in India [32] and France [33]. This was due to the fact that alcohol use decreases awareness and inhibits judgments. In addition, alcohol consumption leads to significant impairment of information processing and motor performance and induces a specific set of physical sensations [34]. Moreover, frequent alcohol consumption results in attention deficient, impulsive behavior, aggressiveness, and intoxication. As a result, patients may have less concern about their own health.

Furthermore, this study showed a significant association between late presentation to HIV/AIDS care and symptoms at the time of HIV diagnosis. Late presentation of those who had symptoms during HIV diagnosis was nearly three times more likely compared to those who had no symptoms. This finding is consistent with those of studies done in central Haiti, Cameroon [23, 24] and Switzerland [35]. This could be explained by the fact that the presence of symptoms at the time of diagnosis could result in identity confusion and hopelessness in a person’s life which in turn affects early healthcare seeking behavior.

Finally, HIV positive adults with chronic illnesses during the time of HIV infection were identified as predictors of late presentation to HIV/AIDS care. According to this study, late presentation of adults with chronic illnesses was nearly 3.5 times more compared to adults who had no such illnesses. This finding was in agreement with that of a study done in Cameroon [24, 31]. The reason may be that adults who had chronic illnesses preferred treatment for the other illnesses primarily. As a result, they presented late to HIV/AIDS care. Moreover, individuals might attend HIV testing and treatment whenever they develop HIV/AIDS-related diseases.

## Conclusion

Late presentation to HIV/AIDS care of adults living with HIV/AIDS was found to be high. High fear of stigma, frequent alcohol use, symptoms at the time of HIV diagnosis, and chronic illnesses were significantly associated with late presentation to HIV/AIDS care. Therefore, we recommend community-based outreach early HIV testing and counseling for all individuals. Early enrollment and linkage to HIV/AIDS care also needs to be strengthened. Furthermore, awareness creation about the problems of late presentation to HIV/AIDS care is important.

## Strength and limitation of the study

I-square shows a significant heterogeneity. Hence, we applied random-effect model and deal with the reasons of heterogeneity. Therefore, the pooled result can be used for policy implication because we checked that the outcome is similar in all studies with binary outcome (being late or not lately initiate ART), similar study participants involved, similar study design use, and all studies are in good methodological quality.

To be sure, this high quality attempt is not free from limitations as it may be subject to the unpublished and unaddressed issues. The result of this work is not representative of all regions since data were not gathered from Afar, Dire Dawa, Addis Ababa, Somali, Gambella and Beshangul Gumuze.

## Additional files

Additional file 1: PRISMA guideline checklist. (DOCX 300 kb)

Additional file 2: Searching strings used for PubMed. (DOCX 12 kb)

## Data Availability

All data generated or analyzed during study included in this systematic review and meta-analysis.

## Abbreviations

AIDS: Acquired Immunodeficiency Virus
AOR: Adjusted Odds Ratio
ART: Antiretroviral Therapy
CI: Confidence Interval
HIV: Human Immunodeficiency Virus
JBI: Joanna Briggs Institute
OR: Odds Ratio
