# Supplementary figures and images for: Late presentation of HIV positive adults and its predictors to HIV/AIDS care in Ethiopia: a systematic review and meta-analysis
Source: BMC Infect Dis. 2019 Jun 17;19:534. doi: 10.1186/s12879-019-4156-3 (PMC6580488; doi:10.1186/s12879-019-4156-3)

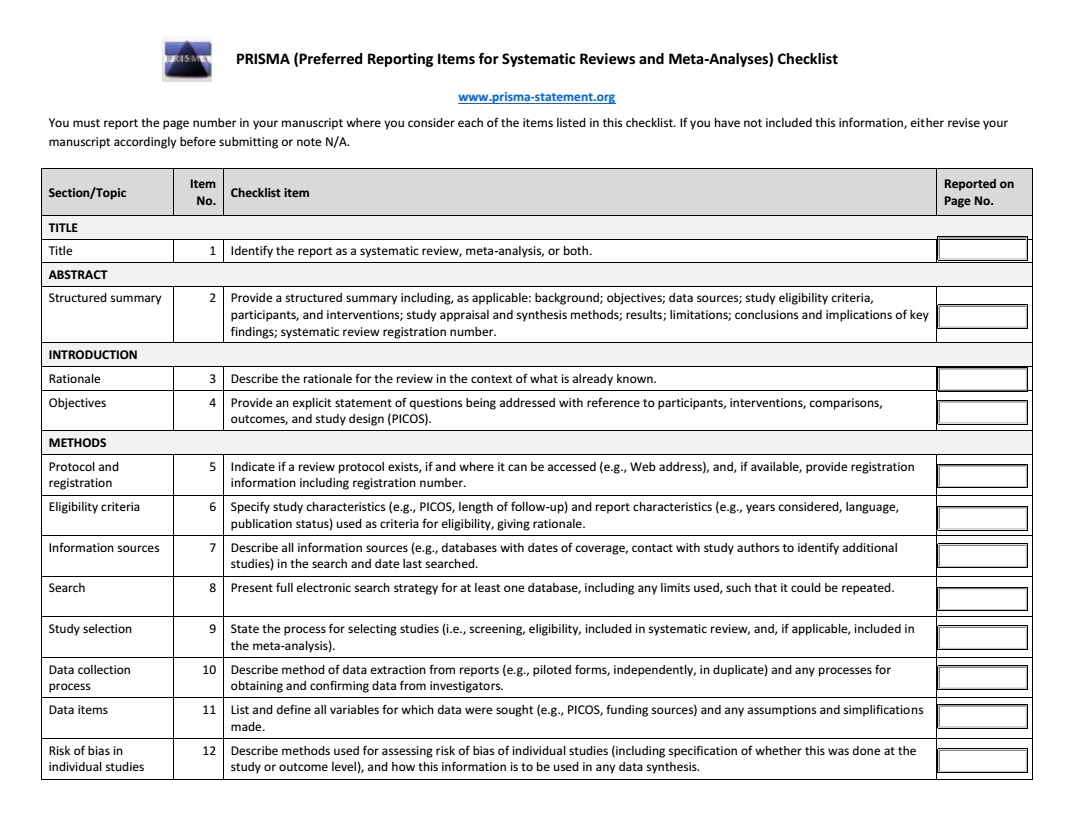


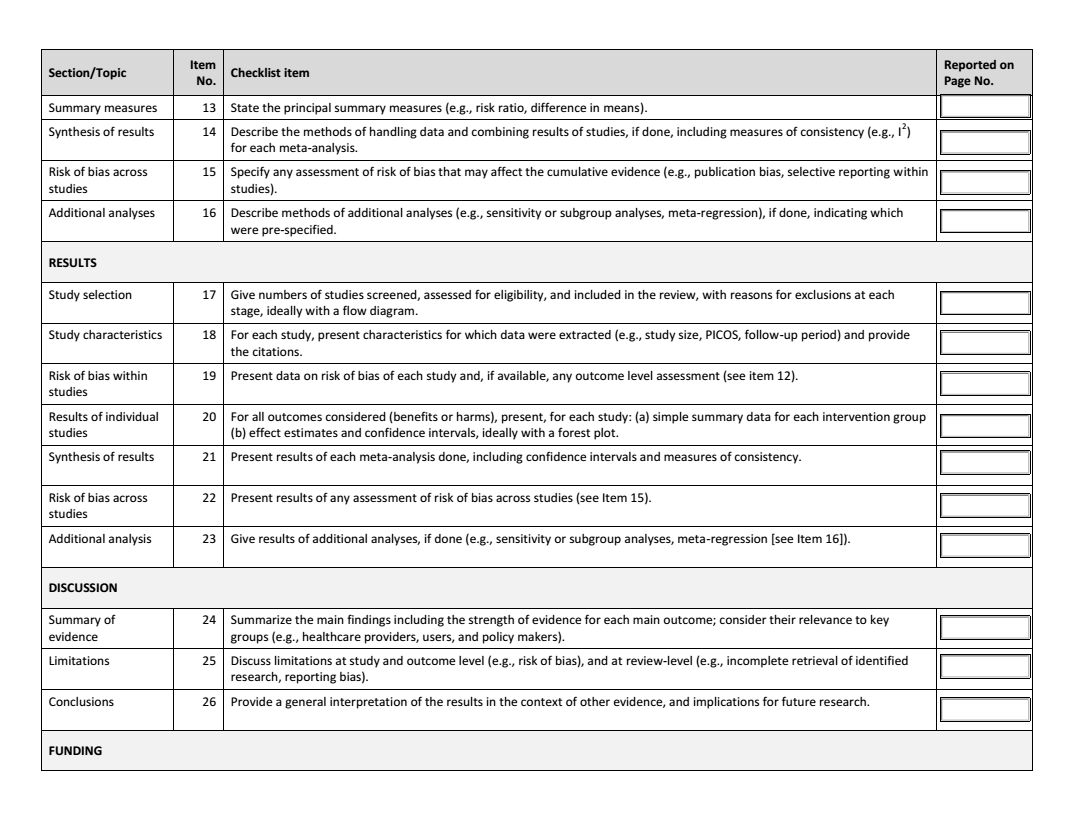

Supplement: Supplementary file 1 — PRISMA guideline checklist. (DOCX 300 kb) [file 12879_2019_4156_MOESM1_ESM.docx]
