# Supplementary material for: Late presentation of HIV positive adults and its predictors to HIV/AIDS care in Ethiopia: a systematic review and meta-analysis
Source: BMC Infect Dis. 2019 Jun 17;19:534. doi: 10.1186/s12879-019-4156-3 (PMC6580488; doi:10.1186/s12879-019-4156-3)
Supplement: Supplementary file 2 — Searching strings used for PubMed. (DOCX 12 kb) [file 12879_2019_4156_MOESM2_ESM.docx]

((Late presentation) OR (late presentation {MeSH terms}) OR (delay presentation) OR (delay presentation{MeSH terms}) OR (advanced stage presentation) OR (advanced stage presentation{MeSH terms}) OR (late stage presentation) OR (late stage presentation {MeSH terms}) AND (Human immune deficiency virus care) OR (Human immune deficiency virus care {MeSH terms}) OR (Human immune deficiency virus/acquired immune deficiency syndrome care) OR (Human immune deficiency virus/acquired immune deficiency syndrome care {MeSH terms}) OR (HIV care) OR (HIV care{MeSH terms}) AND (HIV/AIDS care) OR (HIV/AIDS care{MeSH terms}) AND (associated factors) OR (associated factors{MeSH terms}) OR (predictors) OR (predictors{MeSH terms}) OR (determinants) OR determinants{MeSH terms}) OR (risk factors) OR (risk factors {MeSH terms}) AND (HIV positive individuals) OR (HIV positive individuals {MeSH terms) OR (HIV + adults) OR (HIV+ adults {MeSH terms}) AND (Ethiopia))
